# Supplementary material for: Integrating whole-genome sequencing within the National Antimicrobial Resistance Surveillance Program in the Philippines
Source: Nat Commun. 2020 Jun 1;11:2719. doi: 10.1038/s41467-020-16322-5 (PMC7264328; doi:10.1038/s41467-020-16322-5)
Supplement: Supplementary file 1 — Reporting Summary [file 41467_2020_16322_MOESM1_ESM.pdf]

nature research

Corresponding author(s): Silvia Argimón (submission and review)
David M. Aanensen (after publication)
Celia Carlos (after publication)

Last updated by author(s): Apr 14, 2020

Reporting Summary

Nature Research wishes to improve the reproducibility of the work that we publish. This form provides structure for consistency and transparency in reporting. For further information on Nature Research policies, seeAuthors & Referees and theEditorial Policy Checklist .

Statistics

For all statistical analyses, confirm that the following items are present in the figure legend, table legend, main text, or Methods section.

n/a	Confirmed	
☐	☒	The exact sample size (*n*) for each experimental group/condition, given as a discrete number and unit of measurement
☒	☐	A statement on whether measurements were taken from distinct samples or whether the same sample was measured repeatedly
☐	☒	The statistical test(s) used AND whether they are one- or two-sided<br>*Only common tests should be described solely by name; describe more complex techniques in the Methods section.*
☒	☐	A description of all covariates tested
☒	☐	A description of any assumptions or corrections, such as tests of normality and adjustment for multiple comparisons
☒	☐	A full description of the statistical parameters including central tendency (e.g. means) or other basic estimates (e.g. regression coefficient) AND variation (e.g. standard deviation) or associated estimates of uncertainty (e.g. confidence intervals)
☐	☒	For null hypothesis testing, the test statistic (e.g. *F*, *t*, *r*) with confidence intervals, effect sizes, degrees of freedom and *P* value noted<br>*Give P values as exact values whenever suitable.*
☒	☐	For Bayesian analysis, information on the choice of priors and Markov chain Monte Carlo settings
☒	☐	For hierarchical and complex designs, identification of the appropriate level for tests and full reporting of outcomes
☒	☐	Estimates of effect sizes (e.g. Cohen's *d*, Pearson's *r*), indicating how they were calculated

Our web collection on statistics for biologists contains articles on many of the points above.

Software and code

Policy information about availability of computer code

Data collection

The epidemiological, clinical, and antibiotic susceptibility data in this study is collected with and/or stored in WHONET v5.6, a free Windows-based database software for the management and analysis of microbiology laboratory data.

Data analysis

Ariba v.2.6.1
Bandage v0.8.1
bcftools v0.1.19 (http://www.htslib.org/doc/samtools-0.1.19.html)
BLASTn v2.7.0
BRIG v1.0
Burrows Wheeler Aligner v0.7.12
Circlator v1.5.3 (https://sanger-pathogens.github.io/circlator/)
GapFiller v1.11
Gubbins v1.4.10
MARA (no version provided, https://galileoamr.arcbio.com/mara/)
Microreact (www.microreact.org)
MLSTcheck v1.007001 (https://www.sanger.ac.uk/science/tools/mlstcheck)
Prokka v1.5
Quiver (no version provided, https://github.com/PacificBiosciences/GenomicConsensus)
RAxML v8.2.8
samtools v0.1.19 (http://www.htslib.org/doc/samtools-0.1.19.html)
snp-sites v2.4.1
SSPACE v2.0
Tableau Desktop v2018.3.11
Unicycler v0.4.0

vcfutils.pl (https://github.com/lh3/samtools/blob/master/bcftools/vcfutils.pl)
Velvet v1.2
VelvetOptimiser v2.2.5
WHONET v5.6

For manuscripts utilizing custom algorithms or software that are central to the research but not yet described in published literature, software must be made available to editors/reviewers. We strongly encourage code deposition in a community repository (e.g. GitHub). See the Nature Research guidelines for submitting code & software for further information.

Data

Policy information about availability of data

All manuscripts must include a data availability statement. This statement should provide the following information, where applicable:
- Accession codes, unique identifiers, or web links for publicly available datasets
- A list of figures that have associated raw data
- A description of any restrictions on data availability

Raw sequence data generated during this study have been deposited in the European Nucleotide Archive (ENA) under the study accession PRJEB17615, [https://www.ebi.ac.uk/ena/data/view/PRJEB17615]. Run accessions are provided in the Microreact projects linked throughout the manuscript and below. The plasmid sequence assemblies generated during this study are available from Tables 2 to 4. The resistance database used in this study is available at (https://figshare.com/s/94437a301288969109c2). The multi-locus sequence type databases used in this study are available at PubMLST (https://pubmlst.org) or at BIGSdb (https://bigsdb.readthedocs.io/en/latest/#).
The data presented in this study are available from the following Microreact projects:
https://microreact.org/project/ARSP_ABA_2013-14
https://microreact.org/project/ARSP_PAE_2013-14
https://microreact.org/project/ARSP_KPN_2013-14
https://microreact.org/project/ARSP_ECO_2013-14
https://microreact.org/project/ARSP_KPN_ST340_2013-14
https://microreact.org/project/ARSP_KPN_ST147_2013-14
https://microreact.org/project/ARSP_KPN_ST147_GLOBAL
https://microreact.org/project/ARSP_ECO_ST410
https://microreact.org/project/ARSP_ECO_ST410_GLOBAL

Field-specific reporting

Please select the one below that is the best fit for your research. If you are not sure, read the appropriate sections before making your selection.

☒ Life sciences ☐ Behavioural & social sciences ☐ Ecological, evolutionary & environmental sciences

For a reference copy of the document with all sections, see nature.com/documents/nr-reporting-summary-flat.pdf

Life sciences study design

All studies must disclose on these points even when the disclosure is negative.

Sample size

The bacterial pathogens included in the See and Sequence study were those responsible for the majority of antimicrobial resistant infections in the Philippines, as listed on the Methods. All carbapenemase-producing K. pneumoniae and E. coli isolates referred to, confirmed, and banked by the ARSRL in 2013-2014 were selected for the retrospective sequencing survey. Approximately 100 isolates of carbapenemase-producing P. aeruginosa and A. baumannii each, were selected according to the following criteria: i) referred to ARSRL in 2013-2014; ii) complete resistance profile (i.e., no missing data); iii) overall prevalence of the resistance profile in the ARSP data (including both referred and non-referred isolates); iv) geographical representation of different sentinel sites. The number of isolates included from each sentinel site was proportional to their relative abundance and estimated from (n/N)*100 (rounded up), where n is the total number of isolates from one site, and N is grand total of isolates ; v) when both invasive and non-invasive isolates representing a combination of resistance profile, sentinel site and year of collection were available, invasive isolates (i.e. from blood, or cerebrospinal, joint, pleural and pericardial fluids) were given priority. In addition, approximately 100 isolates of ESBL-producing E. coli and K. pneumoniae each were included as per the criteria above. Pan-susceptible isolates were only available for P. aeruginosa isolates and were also included in the retrospective sequencing survey.

No sample size power calculation was carried out. Strain selection for WGS aimed to represent the diversity of prevalent antibiotic-resistance profiles in the Philippines with an emphasis on key resistances identified by the ARSP as important to public health, and defined by the World Health Organization's global priority list of antibiotic-resistant bacteria to guide research, discovery, and development of new antibiotics. Bacterial strains were isolated from a variety of clinical samples that represent different infection types.

Data exclusions

Bacterial isolates could not be included/were excluded when their resistance profile was incomplete (i.e. one or more antibiotics had not been tested), there was a lack of growth upon resuscitation of lyophilisates, species id did not match the original record, the key resistance phenotype tested did not match the original record. Genomes were excluded when the sequence data did not pass quality control, or the species identification did not match the expected organism. Exclusion criteria based on completeness and concordance of the species id and the resistance profile were pre-established.

Replication

The reproducibility of the results was ensured by a stringent quality control of the sequence data, by including bootstrap replication in the inference of phylogenetic trees (100 replicates) and, when possible, by conducting independent phylogenetic analyses with different methods.

Randomization	Sentinel sites submitting isolates for this study are members of the Antimicrobial Resistance Surveillance Program in the Philippines. Random sampling did not apply to our study, as isolates of each organism were selected as described above.
Blinding	Blinding was not applicable to our study, as it did not include experimental and control groups.

Reporting for specific materials, systems and methods

We require information from authors about some types of materials, experimental systems and methods used in many studies. Here, indicate whether each material, system or method listed is relevant to your study. If you are not sure if a list item applies to your research, read the appropriate section before selecting a response.

Materials & experimental systems

n/a	Involved in the study	
☒	☐	Antibodies
☒	☐	Eukaryotic cell lines
☒	☐	Palaeontology
☒	☐	Animals and other organisms
☒	☐	Human research participants
☒	☐	Clinical data

Methods

n/a	Involved in the study	
☒	☐	ChIP-seq
☒	☐	Flow cytometry
☒	☐	MRI-based neuroimaging
