# Supplementary material for: Integrating whole-genome sequencing within the National Antimicrobial Resistance Surveillance Program in the Philippines
Source: Nat Commun. 2020 Jun 1;11:2719. doi: 10.1038/s41467-020-16322-5 (PMC7264328; doi:10.1038/s41467-020-16322-5)
Supplement: Supplementary file 3 — Peer Review File [file 41467_2020_16322_MOESM3_ESM.pdf]

Editorial Note: This manuscript has been previously reviewed at another journal that is not operating a transparent peer review scheme. The manuscript was considered suitable for publication without further review at Nature Communications.
